# The impact of long‐haul travel and 13 h time change on sleep and rest activity circadian rhythm in speed skaters during World Cup competitions

**DOI:** 10.1113/EP092195

**Published:** 2024-11-01

**Authors:** Giorgio Varesco, Chun William Yao, Evelyne Dubé, Guido Simonelli, François Bieuzen

**Affiliations:** ^1^ National Institute of Sport of Québec Montréal QC Canada; ^2^ Center for Advanced Research in Sleep Medicine Hôpital du Sacré‐Coeur de Montréal, CIUSSS du Nord de l’Île‐de‐ Montréal Montréal QC Canada; ^3^ Department of Medicine Université de Montréal Montréal QC Canada; ^4^ Department of Neuroscience Université de Montréal Montréal QC Canada

**Keywords:** athletes sleep, jet‐lag, long‐haul travels, sport performance

## Abstract

Athletes frequently compete only a few days after long‐haul travel. Longitudinal real‐world data on athletes’ sleep and sleep–wake cycle in competitive settings remain scarce. This study assessed the impact of a long‐haul travel across ∼13 time zones on sleep patterns, rest–activity circadian rhythms (RAR), and their subsequent effects on neuromuscular function and race performance in the Canadian Short‐Track Speed Skating Team. Nineteen athletes (24 ± 4 years, 11 women) travelled from Montréal (UTC‐5) to Asia (UTC+8, UTC+9) for World Cup races between 2017 and 2019. Actigraphy data were collected before (Baseline) and during travel, during the stay in Asia (SIA), and during competition days. RAR were computed using cosinor analyses on accelerometry data with 24 h phase periods. Countermovement jump height (CMJ) was measured in a subsample (*n* = 10). Compared to baseline (7:08 ± 0:53), athletes obtained less sleep during travel (6:16 ± 1:27) and competition days (6:35 ± 1:10), and more during SIA (7:32 ± 0:46; time effect *P *< 0.0001). Sleep efficiency and CMJ were greater in SIA than baseline (*P* = 0.007 and *P* = 0.0004, respectively). During SIA, sleep time increased by 9 min per night until the fifth day (*P *< 0.0001), with a slight decrease in sleep efficiency (*P* = 0.005) and an increase in CMJ (*P *< 0.0001). For RAR, mean activity peaked on day 2, shifting from late evening to ∼15:00. Race performance was not different from other races of the same season (*P *> 0.254). Our results demonstrated that, despite the possible sleep debt from the long‐haul travel, athletes recovered within 5 days, highlighting their adaptability to manage sleep debt and jetlag without impacting competitive outcomes.

## Abstract (highlights)

- **What is the central question of this study?**
  Which are the effects of long‐haul travel crossing ~13 time zones on sleep, RAR and performance in speed skaters travelling for competitions?
- **What is the main finding and its importance?**
  Total sleep time progressively increased for up to 5 days after landing, along with adjustments in RAR. Neuromuscular performance and competition performance remained unaltered, indicating that any potential impaitments due to travel or jet‐lag were resolved within 5 days.

## 1 INTRODUCTION

Athletes competing at the highest level face frequent long‐haul travel to take part in events such as World Cup competitions, World Championships or the Olympic games. Crossing multiple time zones in a short time disrupts the circadian rhythm due to the mismatch between the physiological clock that regulates wake–sleep cycles and the actual external time (i.e., jet lag; Waterhouse et al., 2004). Jet lag after long‐haul travels can persist for several days, during which the circadian rhythm re‐synchronizes progressively to the new local time through psychobiological, behavioural and environmental cues, called ‘zeitgebers’ (Reilly et al., 2005; Waterhouse et al., 2004).

Recently, discussions within the scientific community about the consequences of long‐haul travel have been heightened by the recent summer and winter Olympic and Paralympic Games in Asia, where Western athletes had to cross up to 13 time zones before the competition (e.g., Fagher et al., 2022; Ishikura et al., 2023; Roach, 2019; Rossiter et al., 2022). While it is well established that disturbances in athlete psychobiological state and sleep are associated with poor performance (Fullagar et al., 2015), the effects of long‐haul travels on athletic performance remain to be clarified (Reilly et al., 2005; Rossiter et al., 2022), and studies performed in high‐level competition settings remain rare (e.g., Biggins et al., 2022; Fowler et al., 2017; Lalor et al., 2021; Rossiter et al., 2022). This is mainly due to logistic challenges and the limited access to elite athletes during competitive periods (Janse van Rensburg et al., 2021).

Polysomnography, the gold standard for sleep assessment, provides comprehensive data on sleep architecture but is invasive and time‐consuming, limiting its use in field research. One alternative is the use of actigraphy devices, which provide a non‐invasive method for assessing sleep patterns and circadian rhythm in sporting contexts. For example, using actigraphy, Rossiter et al. (2022) showed that elite swimmers traveling for training and competition tend to sleep more after a −8 h time change, with no changes observed in neuromuscular performance (CMJ jump) or athletic performance at a swimming test after the travel (Rossiter et al., 2022). Conversely, Biggins et al. (2022) found a decrease in sleep duration up to 5 days post‐travel after −7 h time zone change in soccer players. Biggins et al. (2022) did not assess performance or indices of circadian rhythm. Discrepancies across studies could be due to the different disciplines practiced: indeed, previous evidence showed differences across different disciplines in terms of rest–activity circadian rhythm (RAR) matrices (Vitale et al., 2019), which describe the amplitude and shift in physically active behaviour and could serve as markers of the circadian timing system (Lévi et al., 2014; Roveda et al., 2019). Indeed, physical activity is a potent zeitgeber (Barger et al., 2004; Lewis et al., 2018), but while actigraphy has been extensively used to capture sleep patterns in athletes, the use of diurnal physical activity data to compute RAR matrices in sport settings remains scarce compared to clinical ones (Ancoli‐Israel et al., 2003; Vitale et al., 2019).

In the context of traveling for competitions, the evolution of the distribution of RAR matrices could be a relevant indicator of circadian rhythm adjustment that integrates the effect of jet lag with the important workload athletes are exposed to (Rossiter et al., 2022; Waterhouse et al., 2002). To the best of our knowledge, no study has measured the evolution of RAR after long‐haul travel in athletes.

In the context of the present study, we evaluated actigraphic data, neuromuscular performance, workload and competition performance data of the Canadian short‐track speed skating National Team. Speed skating involves multiple distances ranging from 500 to 1500 m, with durations of physical effort from less than 40 s (500 m) up to 150 s (1500 m). Usually, athletes undergo multiple rounds of competition, including qualification rounds, heats, quarterfinals, semifinals and finals. These stages are distributed across a race weekend, with qualification rounds usually held on Fridays and main tournament rounds on Saturdays and Sundays. Skaters might compete in multiple events per day. To win the race, athletes must develop maximal propulsive power over extended periods while also maintaining focus on the technical and tactical aspects of the competition (Bullock et al., 2008; Felser et al., 2016). Both the neuromuscular and cognitive functions are heavily stressed during the performance and could be importantly impacted by a lack of sleep and fatigue. Finally, considering the high level of Asian nations in short‐track speed skating, it is not unusual for World Cup events to be held in Asia. Consequently, Canadian athletes usually need to cross approximately 12 to 14 time zones 1 week before competing, as they did for the past two Winter Olympic Games (Beijing 2022; Pyeongchang, 2018). However, no data on sleep in short‐track speed skaters (or similar sports) during competitive periods are available.

This study aimed to assess sleep duration and efficiency, and to explore the evolution of rest–activity circadian rhythms (RAR) in the Canadian Short‐Track Speed Skating Team competing in countries with ∼13 h of time difference. We hypothesized that less sleep would be obtained during travel (Lastella et al., 2019; Rossiter et al., 2022) and sleep time would recover to baseline before the competition, that is, within 5 days from landing (Biggins et al., 2022). We also hypothesized that neuromuscular performance would decrease following travel, recovering quickly until the day of the competition. Importantly, the authors were aware of the results of the competition before the beginning of the data analysis, and thus a hypothesis on performance would likely be biased and was therefore not formulated.

## 2 METHODS

### 2.1 Participants

Nineteen elite short‐track speed skaters from the Canadian National Team (11 females, age: 24 ± 4 years; height: 169 ± 0.06 m; body mass: 64.4 ± 7.3 kg) participated and travelled from Montréal (UTC‐5) to Asia (UTC+8, UTC+9) for World Cup races between 2017 and 2019. The sample included eight athletes who had won at least one Olympic medal, and another five athletes who had won at least one World Championship Medal.

Races in 2017 were held in Shanghai (China, UTC+8) and Seoul (South Korea, UTC+9). Races in 2019 were held in Nagoya (Japan, UTC+9) and Shanghai. Races took place on two consecutive weekends, during which each athlete competed in multiple events: 14 athletes competed in the 500 m events, 12 athletes in the 1000 m events and 10 athletes in the 1500 m events.

The data collection presented in this study was part of the procedures performed by the National Institute of Sport Québec (Canada) to prepare for the 2022 Olympic Games held in China. Elite skaters provided written consent, and data collection was carried out respecting provincial legislation and conformed to the standard set by the *Declaration of Helsinki* (2013). The present analysis was conducted as part of a larger project aiming at investigating sleep in athletes and was approved by the Ethical Research Committee of the CIUSSS du Nord‐de‐l’Île‐de‐Montréal (no. 2024–2718).

### 2.2 Study design

**FIGURE 1:**
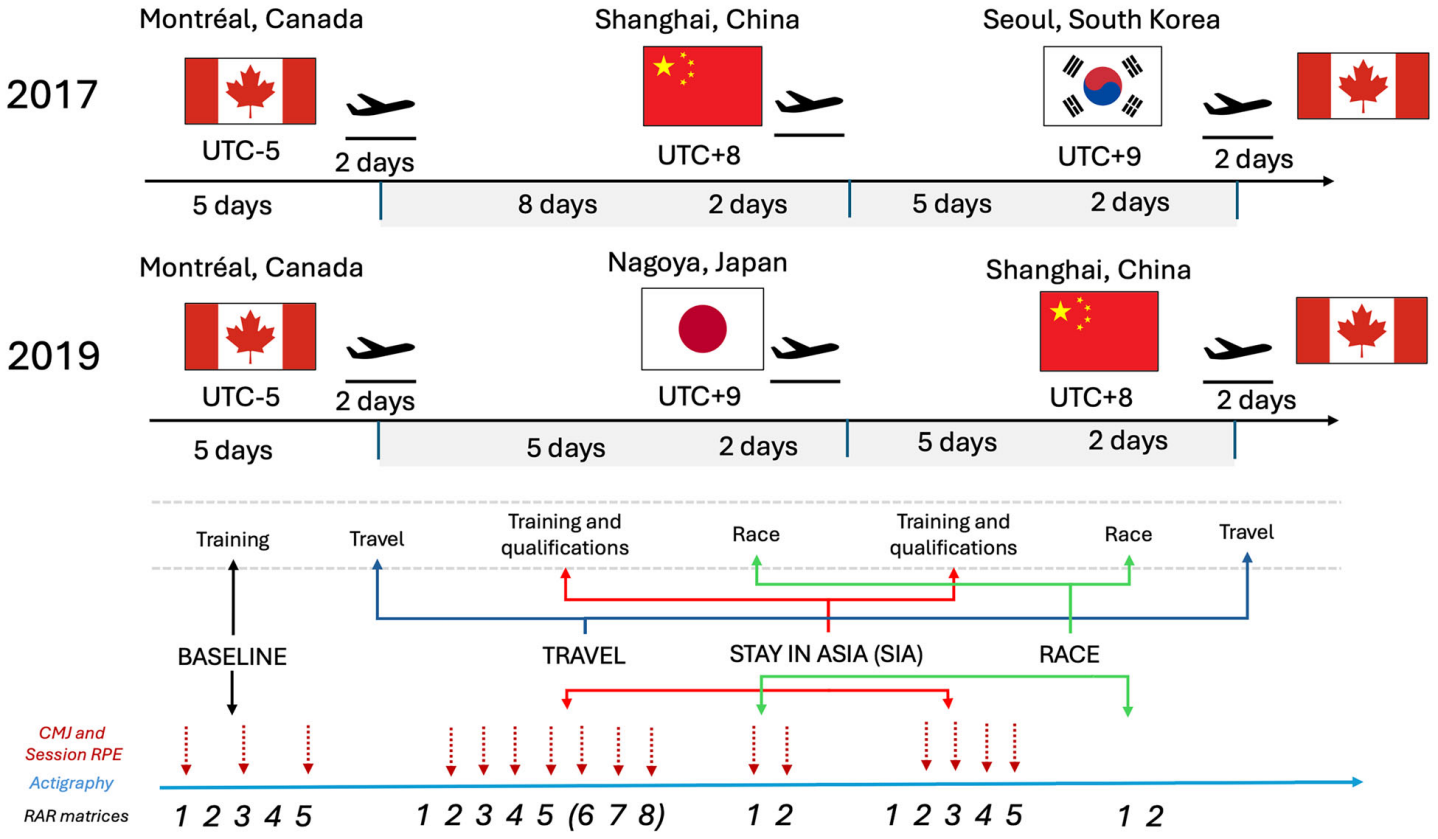
Description of the different phases of the competition periods evaluated in the present study. The duration of the phases of training, qualification and race in Asia are presented in the grey areas. Codification of the different time phases for the analysis and time points of the measures collected are presented at the bottom. ‘Travel’ indicates long haul travel with an important time shift. As such, travel across cities in Asia was not included. Red dotted arrows represent countermovement jump height (CMJ) and rate of perceived exertion (RPE) session data collection. Actigraphy data (blue arrow) were monitored continuously, while rest–activity rhythm (RAR) matrices were not computed during travel days (black numbers indicate the day of a given time period).

The study employed a prospective observational design with non‐invasive procedures. In both 2017 and 2019, speed skaters were required to wear a wrist‐worn actigraphy device (Motionwatch 8, CamNtech Ltd, Fenstanton, UK) continuously from 1 week before international travel to Asia until their return to North America to capture sleep patterns and RAR matrices during key phases: Baseline (5 days), Travel (4 days), Stay in Asia (SIA, 13 days in 2017 and 10 days in 2019) and Race (4 days) during the 2017 and 2019 World Cup (Figure 1). In 2017, during SIA, days 5−8 (Figure 1) were approximated to day 5, after verifying that the results were not affected. Successively, data from 2017 and 2019 were pooled together in a single data frame for statistical analysis. During the study, athletes were free to manage their sleep schedule but were required to wake up before 09:00 h to consume breakfast. Additionally, athletes did not consume any drugs or supplements to facilitate sleep, nor did they receive ad hoc sleep hygiene education. Other measures recorded included session rate of perceived exertion (RPE) of training (Foster et al., 2001; Haddad et al., 2017), neuromuscular data obtained from countermovement jumps (CMJs), and race performance (Figure 1). Neuromuscular data and session RPE were collected only in a subgroup of athletes (*n* = 10) before the training or race.

### 2.3 Actigraphic data

Athletes wore a Motionwatch 8 actigraph on the non‐dominant wrist. The device recorded data over 30 s‐long epochs and the threshold for sensitivity to sleep was set as high using the proprietary software (Motionware v1.3; CamNtech). Sargent et al. (2016) previously showed these settings to have good agreement, specificity and sensitivity in estimating total sleep time when compared with polysomnography in elite athletes. Elite speed skaters were familiar with wearing actigraphy monitors and were instructed to try not to cover the light sensor with clothes around bedtime and wake‐up time, defined as the ‘subjectively reported behavioural indicators reflecting the time (hh:mm:ss) a person chooses to start trying to fall asleep and conclude their attempts to sleep, respectively’ (De Zambotti et al., 2024). Thus, skaters were instructed to press the event marker button on the device at both bedtime and wake‐up time. The button sets a temporal marker when exporting data. This temporal marker and the light sensor data were used to check bedtime and wake‐up time estimated by the software's algorithm. When exporting, sleep was manually aligned with local time (i.e., Montréal, Seoul, Shanghai or Nagoya). Data during Travel were not reliable for bedtime and wake‐up time (being absolute time stamps) and thus excluded. Sleep efficiency was also computed as the percentage of time spent asleep while in bed, compared to the total time spent in bed (De Zambotti et al., 2024; Elbaz et al., 2012). Total sleep represents sleep obtained during night‐time as we did not observe daytime naps in the present data.

### 2.4 Rest‐activity circadian rhythms and cosinor analysis

Accelerometry‐based data were used to compute RAR variables, as previously described for elite soccer, volleyball and triathlon athletes (Vitale et al., 2019). In detail, the tri‐axial accelerometer of the Motionwatch sampled continuously at 50 Hz, with a bandpass filter of 3–11 Hz. This filtering was set by the manufacturer to remove gravitational artifacts at the frequencies <3 Hz and shock/vibration artifacts at frequencies >11 Hz. The peak accelerations recorded over 1 s and >0.1G are then summed over the 30 s epochs. These values are then scaled by the Motionwatch 8 onboard software to produce activity counts (AC) for each epoch. As for actigraphy data, AC were manually aligned with local time. A cosine function was fit to AC data via linear cosinor modelling using the card package (Shah, 2020) in the R environment. Extracted variables were (i) the rhythm‐adjusted mean over 24 h (MESOR), (ii) the amplitude of the rhythmic cycle variation, and (iii) the acrophase, that is, the time from midnight to the estimated peak value (Ancoli‐Israel et al., 2003). To avoid the disturbances to data computation induced by hour change and travel, actigraphy recordings of the travel day from North America to Asia were omitted.

### 2.5 Neuromuscular data

The athletes performed a set of three CMJs (separated by ∼10 s) to assess impulse (i.e., area under the curve) before daily skating practice. CMJs were performed with hands on the hips. CMJ data were collected using a portable force plate (PS‐2142, Pasco, Roseville, CA, USA). The highest impulse value recorded for each set was retained for further analyses. Jump height derived from impulse was then computed and retained for statistical analyses. CMJ height data were not collected during Travel and the first day of SIA.

### 2.6 Session RPE

Session RPE was collected after every ice‐skating training at Baseline and during SIA. RPE was scored on a modified CR‐10 Borg scale (Foster et al., 2001) and computed as Training Load (AU) = RPE (AU) × session duration (min).

### 2.7 Race performance

Best race time and ranking were collected for the races in Asia and compared with the race data of the other competitions of the seasons 2017–2018 and 2019–2020 to check for eventual consistent within‐subject underperformance that could be due to a possible effect of the long‐haul travel.

### 2.8 Statistical analysis

A first analysis was performed to evaluate the effect of the different time phases on the studied variables. Total sleep and sleep efficiency were compared across Baseline, Travel, SIA and Race. Bedtime, wake‐up time, RAR variables and CMJ height were compared across Baseline, SIA and Race.

The second analysis estimated the evolution of the primary and secondary outcomes during SIA. Initial visual inspection revealed a rise in sleep time followed by a plateau at day 5 of SIA, prompting further empirical investigation. Fitting a piecewise regression model (segmented package) from day 1 to day 10 of SIA (excluding race days), we confirmed a significant breakpoint at day 5, that is, the day preceding the first race (see Results). Consequently, the analysis was restrained to the period from days 1 to 5 of SIA, ensuring an accurate representation of the evolution of sleep, RAR, workload and neuromuscular variables following the long‐haul travel.

For both analyses, linear mixed‐effects models (LMM, lme4 package) were fitted to the data using the restricted mean likelihood method in the lme4 package to evaluate the overtime changes of the sleep variables (sleep, sleep efficiency, bedtime, wake‐up time), RAR variables (MESOR, amplitude, acrophase) and CMJ height. Similar LLM were implemented to compare performance at the different races of the 2017–2018 and 2019–2020 seasons, separating race distances. To interpret RAR matrices in relation to the workload (Vitale et al., 2019), MESOR, acrophase and amplitude were inserted in a LMM as predictors of session RPE.

For all LMM, analysis of deviance (type II Wald's χ^2^ test) was used to evaluate the global main effects of time or day for the first and second analysis, respectively (car package). *P*‐values were extracted from *F*‐tests using Satterthwaite's degrees of freedom method (lmerTest package). Given the dependence of the data for the participants, a random intercept for participants was built into each LMM. The empirical test of the model assumptions was performed via model residuals graphical analysis of the Q–Q plots. Finally, robust LMM were fitted to confirm the results of the first analysis (robustlmm package), limiting the effect of possible extreme values across time phases on the results. When a significant main effect or interaction was observed, Tukey's *post hoc* correction was applied to pairwise comparisons (emmeans package). Estimates from the models and standard errors were calculated using the least‐squares means method. The significance threshold was set at α = 0.05. Statistical analyses were carried out in the R statistical environment (V4.2.3, R Foundation for Statistical Computing, Vienna, Austria). Observed data are presented in the text and expressed as means ± SD.

## 3 RESULTS

Detailed step‐by‐step computation with complete results is available on the Open Science Framework webpage dedicated to the present project (see Data Availability Statement; doi: 10.17605/OSF.IO/QSZ9G). Athletes competed in multiple events across various distances during the races considered for the present study, resulting in a total of 26 participations in 500 m events, 23 participations in 1000 m events, and 28 participations in 1500 m events. Model estimates for sleep parameters, RAR and CMJ are presented in Table A1.

### 3.1 Sleep duration

**FIGURE 2:**
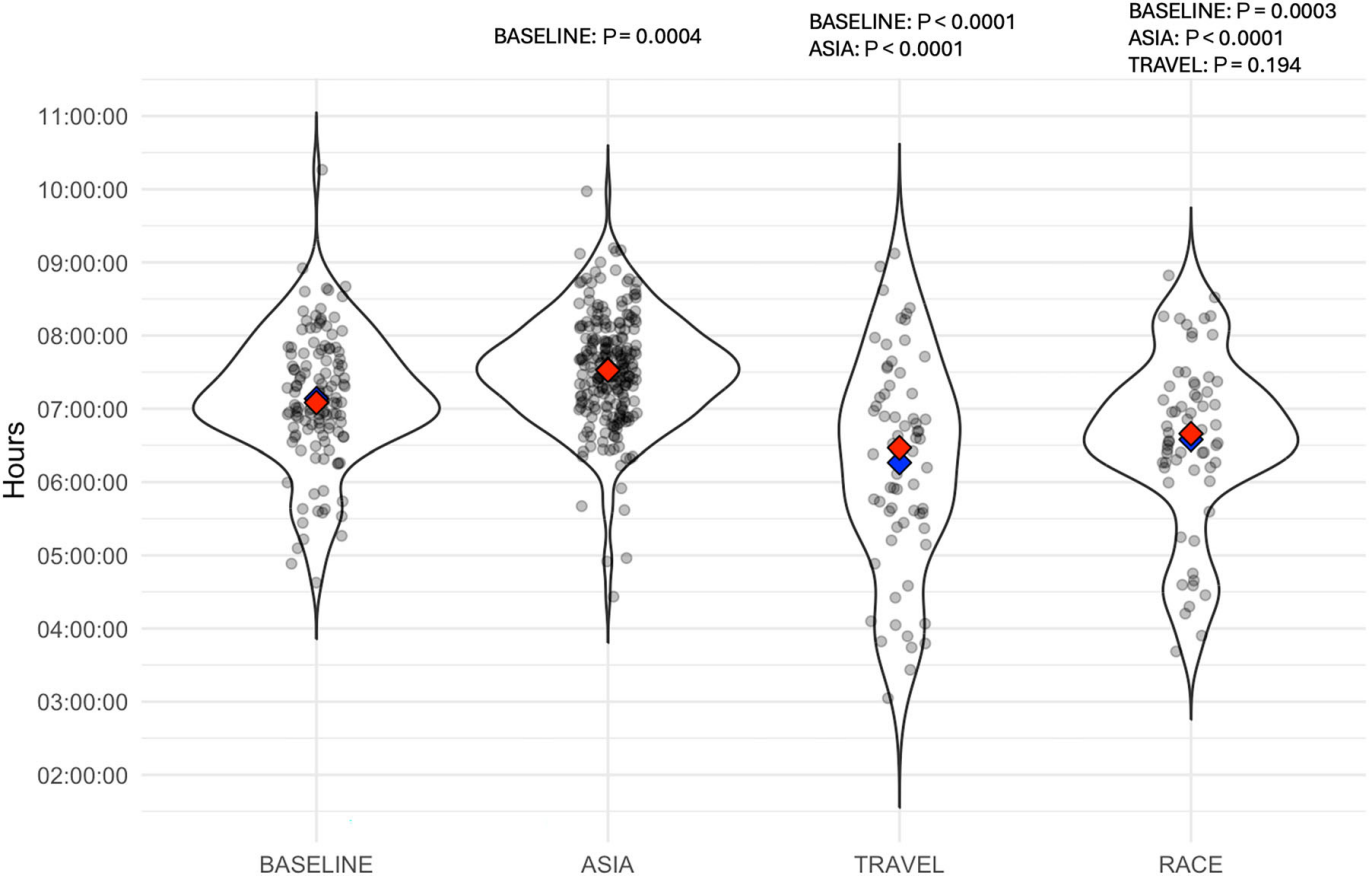
Violin plots of the total sleep obtained by athletes during the different phases of the study. Red diamonds represent the mean, while blue diamonds the median. *P*‐values from pairwise comparison are presented on the top of the figure.

Total sleep time showed a time effect across phases (*P* < 0.0001; Figure 1). Athletes slept more during SIA compared to Baseline (*P* = 0.0004), Travel (*P* < 0.0001), and Race (*P* < 0.0001; Table 1 and Figure 2). Additionally, sleep was reduced during both Travel and Race compared to Baseline (*P* < 0.0001 and *P* = 0.0003, respectively). While the non‐robust model found no difference between race and travel (*P* = 0.194), the robust model detected more sleep during Race (23 ± 9 min, 95% CI: 1; 45 min, *P* = 0.038).

**TABLE 1:** Contrast model estimates for sleep, RAR variables and neuromuscular performance.

	Baseline–SIA		Baseline–Race		Baseline–Travel		SIA–Race		SIA–Travel		Race–Travel	
	β ± SE	95% CI	β ± SE	95% CI	β ± SE	95% CI	β ± SE	95% CI	β ± SE	95% CI	β ± SE	95% CI
Sleep time (min)	−24 ± 6	−40; −9	34 ± 8	12; 55	53 ± 8	31; 74	58 ± 8	38; 78	78 ± 8	57; 97	19 ± 10	6; 43
Sleep efficiency (%)	−1.5 ± 0.5	−2.6; 0.3	−0.5 ± 0.6	−2.1; 1.1	0.4 ± 0.6	−1.1; 2	1 ± 0.6	−0.5; 2.4	1.9 ± 0.6	0.4; 3.3	0.9 ± 0.7	0.9; 2.8
Bedtime (min)	17 ± 6	2; 31	−72 ± 9	−92; −52	—	—	−89 ± 8	−108; −70	—	—	—	—
Wake‐up time (min)	−1 ± 7	−16; 15	−27 ± 9	−48; −6	—	—	−26 ± 8	−46; −7	—	—	—	—
MESOR (AC)	7.4 ± 6.9	−8.8; 23.6	31.7 ± 9.8	8.7; 54.7	—	—	24.3 ± 8.9	3.4; 45.2	—	—	—	—
Acrophase (min)	−38 ± 21	−88; 12	−33 ± 30	−103; 38	—	—	5 ± 27	−59; 70	—	—	—	—
Amplitude (AC)	18.4 ± 7.9	−0.2; 36.9	36.6 ± 11.2	10.2; 63	—	—	18.2 ± 10.2	−5.7; 42.2	—	—	—	—
CMJ height (m)	−0.033 ± 0.01	−0.05; −0.01	−0.04 ± 0.01	−0.07; −0.02	—	—	−0.01 ± 0.01	−0.03; 0.01	—	—	—	—

*Note*: Estimates are calculated using the least‐squares means method. Abbreviations: AC, activity counts; SIA, period of stay in Asia.

**FIGURE 3:**
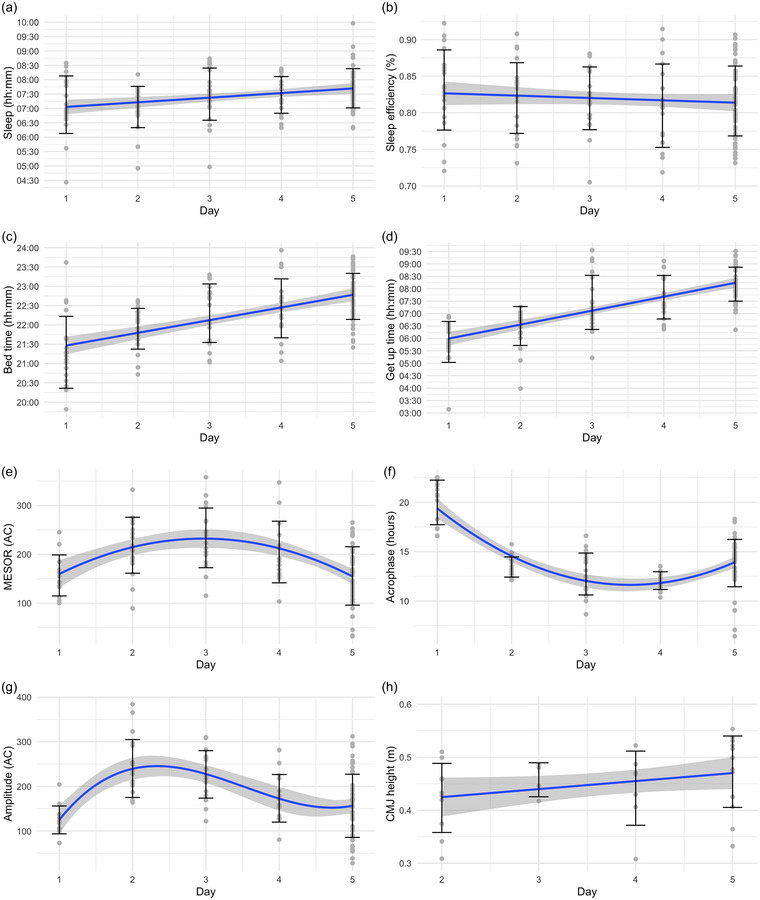
The evolution of sleep time (a), sleep efficiency (b), bedtime (c), wake‐up time (d), MESOR (e), acrophase (f), amplitude (g) and CMJ height (h) from the arrival in Asia (day 1) until the day preceding the race (day 5). Error bars represent the SD for each day. Blue lines represent the estimation from the models (±SE). CMJ height measure was not performed on day 1.

**FIGURE A1:**
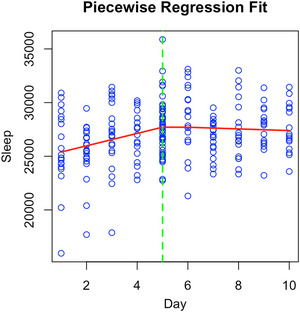
Estimated effect for the piecewise model (red line) used on total sleep data expressed in seconds during SIA (days 1–10). The model identified a breaking point at day 5 (dotted green line; *P* = 0.0002).

A piecewise model assessing sleep during SIA (days 1–10) revealed a day effect with a breaking point at day 5 (*P* = 0.0002, Fig. A1). Separate linear models for days 1–5 and days 5–10 indicated a day effect only in the first period (*P* = 0.0002 vs. *P* = 0.852; Figure 3a), with total sleep increasing linearly by ∼9 min per night (Table 2), likely contributing to the significant differences observed between the phase means.

**TABLE 2:** Model outputs estimating the trajectory of sleep, RAR variables and neuromuscular performance during SIA.

	Intercept				Day (1, 5)				
	β ± SE	95% CI	*t* (df)	*P*‐value	Fit	β ± SE	95% CI	*t* (df)	*P*‐value
Sleep time (hh:mm)	06:57 ± 00:10	06:37; 07:17	41 (97)	<0.0001	*y*∼*x*	00:09 ± 00:02	00:05; 00:14	4 (122)	0.0002
Sleep efficiency (%)	83.8 ± 1.1	81.6; 86	75 (58)	<0.0001	*y*∼*x*	−0.5 ± 0.2	−1; −0.1	−2 (118)	0.017
Bedtime (hh:mm)	21:15 ± 00:10	20:56; 21:34	132 (65)	<0.0001	*y*∼*x*	00:17 ± 00:02	00:13; 00:21	8 (118)	<0.0001
Wake‐up time (hh:mm)	05:31 ± 00:11	05:09; 05:53	30 (89)	<0.0001	*y*∼*x*	00:32 ± 00:03	00:27; 00:37	13 (121)	<0.0001
MESOR (AC)	184 ± 12	160; 207	16 (16)	<0.0001	*y*∼*x* ^2^ *	−120 ± 43^a^	−204; ‐37	−3 (98)	0.006
						−387 ± 42^b^	−469; −304	−9 (98)	<0.0001
Acrophase (hh:mm)	14:04 ± 00:20	13:22; 14:44	41 (15)	<0.0001	*y*∼*x* ^2^ *	−18:31 ± 02:02^a^	−22:28; −14:26	−9 (98)	<0.0001
						20:32 ± 02:01^b^	16:37; 24:33	10 (98)	<0.0001
Amplitude (AC)	179 ± 11	157; 200	17 (17)	<0.0001	*y*∼*x* ^3^ *	−75 ± 53^a^	−178; 27	−1 (98)	0.155
						−418 ± 52^b^	−519; −316	−8 (98)	<0.0001
						242 ± 50^c^	145; 340	5 (96)	<0.0001
CMJ height (m)	0.38 ± 0.03	0.32; 0.45	12 (9)	<0.0001	*y*∼*x*	0.01 ± 0.004	0.007; 0.02	4 (28)	0.0005

*Note*: *Model fitted using orthogonal polynomials. ^a^Linear component. ^b^Quadratic component. ^c^Cubic component.

### 3.2 Sleep efficiency

Sleep efficiency displayed a time effect (*P* = 0.0006). Sleep efficiency was higher during SIA (82.31 ± 4.74%) compared to Baseline (80.99 ± 4.8%, *P* = 0.007) and Travel (80.36 ± 6.81%, *P* = 0.005, Table 1). However, the robust model did not confirm the difference between SIA and Travel (*P* = 0.083). No difference was observed between Race (81.48 ± 4.9%) and Baseline (*P* = 0.846), SIA (*P* = 0.333), or Travel (*P* = 0.534, Table 1). Sleep efficiency decreased slightly over time during SIA (*P* = 0.017; Figure 3b and Table 2).

### 3.3 Bedtime and wake‐up time

A significant time effect was observed for both bedtime (*P* < 0.0001) and wake‐up time (*P* = 0.004). Athletes went to bed later during Race (00:05 ± 01:21 hh:mm) compared to Baseline (22:52 ± 01:00, *P* < 0.0001) and SIA (22:36 ± 00:56, *P* < 0.0001, Table 1). Bedtime was also later at Baseline than during SIA (*P* = 0.019). Similarly, wake‐up times were later during Race (08:10 ± 01:11) compared to Baseline (07:42 ± 00:53, *P* = 0.009) and SIA (07:43 ± 01:07, *P* = 0.005), with no differences between Baseline and SIA (*P* = 0.995). During SIA, bedtime and wake‐up time both increased linearly over time (all *P* < 0.0001; Figure 3c, d). Wake‐up time increase rate was ∼88% steeper than bedtime, which resulted in more time spent in bed over time, explaining the increase in total sleep time (Table 2).

### 3.4 Rest–activity circadian rhythm matrices

A time effect was observed for MESOR (*P* = 0.005) and amplitude (*P* = 0.003) but not acrophase (*P* = 0.195). MESOR was lower during Race (144 ± 74 AC) than both Baseline (186 ± 74 AC, *P* = 0.004) and SIA (175 ± 68 AC, *P* = 0.018), with no difference between Baseline and SIA (*P* = 0.053, Table 1). Both MESOR and acrophase showed a quadratic day effect (*P* < 0.0001), indicating a parabolic relationship over time during SIA (Figure 3e,f).

Amplitude was also lower during Race (142 ± 93 AC) compared to Baseline (188 ± 77 AC, *P* = 0.003), with no difference between SIA and either Baseline (*P* = 0.053) or Race (*P* = 0.174). Over time during SIA, amplitude exhibited a day effect (*P* < 0.0001), best captured by a third‐degree polynomial model (Figure 3g).

### 3.5 Neuromuscular performance

Athletes jumped higher during Race (0.45 ± 0.07 m) compared to Baseline (0.40 ± 0.06 m, *P* = 0.001), with no significant difference between SIA (0.44 ± 0.07 m) and Race (*P* = 0.397). However, CMJ height was greater during SIA than Baseline (*P* = 0.0004), and it increased linearly over time during SIA (*P* = 0.0005; Figure 3h), likely contributing to the significant differences observed between phases.

### 3.6 Session RPE

Session RPE was on average greater at Baseline (4153 ± 1521 AU) than SIA (3539 ± 1273 AU, *P* = 0.003). The intercept of the model was 5768.34 AU (SE: ± 922 AU; *P* < 0.001). A significant negative effect of MESOR on session RPE was observed (β ± SE = −6.63 ± 3.17 AU; *P* = 0.038), indicating that higher MESOR values were associated with lower session RPE. In contrast, no significant effects were found for acrophase (β ± SE = −82.82 ± 53.99 AU; *P* = 0.127) or amplitude (β ± SE = 3.23 ± 3.18 AU; *P* = 0.312). During SIA, no significant day effect was found for session RPE during SIA (*P* = 0.421).

### 3.7 Race performance

**FIGURE A2:**
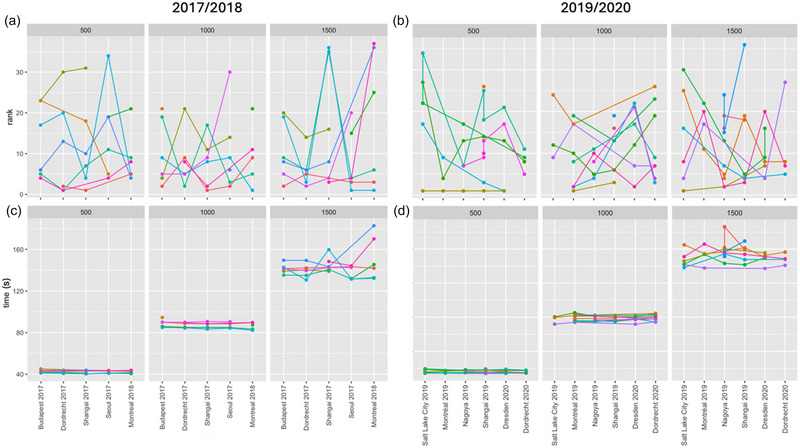
Race results (upper panels) for the season 2017–2018 (a) and 2019–2020 (b) for the athletes recruited in the present study (i.e., taking part in the races in Asia). Bottom panels present the best time performance for each race of the season 2017–2018 (c) and 2019–2020 (d). Different grey panels represent different distances (i.e., 500 m; 1000 m and 1500 m) where short track speed skaters competed. Different colours represent different athletes.

No differences were found in athletes’ race times or rankings across all the 2017–2018 and 2019–2020 seasons for the distances of 500 m (*P* = 0.876), 1000 m (*P* = 0.837) or 1500 m (*P* = 0.254). Detailed race performance data are available in Fig. A2.

## 4 DISCUSSION

This study evaluated sleep, rest‐activity circadian rhythms and performance in the Canadian Short‐Track Speed Skating Team competing 1 and 2 weeks after long‐haul travel involving a ∼13 h local time difference. We observed that less sleep was obtained during the travel compared to baseline, increasing progressively in the 5 days following the arrival to destination. The increased total sleep time was accompanied by a concomitant and progressive adjustment in bedtime, wake‐up time and rest‐activity circadian rhythm parameters. There was no alteration in neuromuscular performance at 2 days following the travel, and performance at the competitions was similar to other competitions performed in the same season.

One of the main strengths of this study was the ability to measure athletes' sleep in a competitive setting. Furthermore, this is the first study to model the evolution of sleep parameters with very large time shifts (i.e., ∼13 h) in athletes. At baseline, speed skaters obtained an amount of sleep comparable to previous data from 11 short‐track speed skaters of the British national team (Leeder et al., 2012). Similarly to what was previously reported (Fullagar et al., 2016; Lastella et al., 2019; Leduc et al., 2021; Rossiter et al., 2022), sleep time was reduced during travel, which could result in sleep debt that athletes might need to recover before the competition (Janse van Rensburg et al., 2021). Indeed, athletes slept more during SIA than at baseline. Conversely, no increase in sleep time was observed in soccer players crossing four time zones westward (Fullagar et al., 2016), while soccer players crossing seven time zones eastward obtained less sleep time during the period following the travel (Biggins et al., 2022). Differences across studies remain to be elucidated and can be due to different travel conditions (Lalor et al., 2021), sleep hygiene awareness (Leduc et al., 2021), and/or different impacts on sleep between eastward versus westward travels (Fowler et al., 2017). However, when crossing ∼13 time zones as in the case of the present study, athletes travel to the other side of the globe and face a complete reversal of the day–night cycle, making travel direction potentially less relevant.

Interestingly, athletes did not compensate for their sleep debt in the immediate days following travel, as might be expected (Fowler et al., 2017; Rossiter et al., 2022). Instead, they progressively increased their sleep duration by approximately 9 min per night, starting from near‐baseline levels on the first night of SIA and reaching an additional hour the night before the competition. A similar increase in sleep time was suggested in elite rowers traveling westward across five time zones, although only two nights were monitored (Kölling et al., 2017). The reasons of this progressive increase in sleep time are unclear and could be due to a combination of residual stress from the travel and adjustments in bedtime and wake time in adaptation to the new local time. By offering sufficient sleep opportunities without imposing a bedtime schedule, we observed that speed skaters went to bed significantly earlier after their arrival in Asia compared to baseline (∼1 h and 15 min), and postponed bedtime each day by ∼17 min until day 5. Wake‐up time occurred earlier after the arrival in Asia compared to baseline (∼1 h and 40 min) but shifted by half an hour per day. The increased sleep opportunity was accompanied by greater sleep efficiency compared to baseline, which can both rule out sleep difficulties consequent to the travel and partially confirm that there was a sleep debt to pay off, as previously observed in non‐competitive settings (e.g., Biggins et al., 2022; Lastella et al., 2019; Rossiter et al., 2022). Conversely, the later bedtimes during race days consequent to evening competitions did not result in extended sleep in the days that followed, consistent with previous data (Fullagar et al., 2016; Lastella et al., 2019).

Estimated MESOR and peak activity were lower during Race compared to both Baseline and SIA, likely due to athletes reducing their ‘out‐of‐ice’ physical activity to conserve energy for competition. Furthermore, after controlling for other RAR metrics, a negative association was found between MESOR and workload, that is, the greater the on‐ice workload, the less active athletes were off‐ice. This suggests that, as could be expected, actigraphy activity counts are inadequate for measuring on‐ice workload, contrary to what was previously suggested for soccer, volleyball or triathlon (Vitale et al., 2019), but they allowed us to assess the spontaneous physical activity (e.g., walking, climbing stairs, etc.) during SIA through RAR metrics (Lévi et al., 2014; Roveda et al., 2019). During SIA, we observed no changes in workload but a parabolic and cubic evolution of estimated mean activity and activity peak, respectively. Both on‐ice workload and estimated spontaneous activity (MESOR and amplitude) were ∼15% greater at Baseline than during SIA. This could be a consequence of tapering strategies before the competition, that is, a progressive decrease in training volume and intensity to maximize recovery for the competition day in favour of maximal performance (Méline et al., 2019). Nevertheless, these results also reflect a prioritization of recovery by athletes for the competition (Fullagar et al., 2023). Interestingly, the acrophase shifted from evening the first day (∼19:00 h) to early‐ mid‐afternoon (∼13:00 h) in the following days, which is closer to what was observed at Baseline. These results provide unique insights into the spontaneous activity level and timing that, together with training, played a key role in providing adaptation to the new local time. Indeed, physical activity is one of the most important non‐photic zeitgebers, as shown by previous experimental studies in non‐athletes and dim‐light laboratory conditions (Okamoto et al., 2013; Yamanaka et al., 2015).

Based on results from this and previous studies evaluating the effects of ∼12 h time changes on sleep and performance (Bullock et al., 2008; Cardinali et al., 2002; Leduc et al., 2021), athletes seem to adapt quickly (i.e., within 5 days), while the literature typically suggests 0.5–1 day of adjustment per time zone crossed (Eastman & Burgess, 2009). Factors such as sufficient sleep opportunities, optimized nutritional regimen and training schedule during the athletes’ time abroad likely contributed to minimizing the impact of travel‐induced fatigue and to rapid adjustments with local time (Janse van Rensburg et al., 2021). In light of the present results, this seems to be more important than other photic or non‐photic interventions such as timed light exposure or melatonin oral consumption (Cardinali et al., 2002). To partially support these claims, results from the CMJ height in a subgroup of athletes exclude the presence of residual travel‐induced fatigue on the day of the competition. Interestingly, CMJ performance increased during SIA and was the greatest in competition days. This is in line with previous observations in swimmers (Rossiter et al., 2022), and could be due to an effect of tapering or the arousal derived by the competition. Furthermore, as for Rossiter et al. (2022) and Bullock et al. (2008), we did not observe any difference in sport‐specific performance.

### 4.1 Limitations

The present study presents some limitations. First, we do not have an objective measure of training load or training‐related fatigability that would allow us to better understand the relationship between training, sleep and circadian rhythm. Second, we did not assess subjective sleep, which limits the comparison of the results from the current and previous studies to objective measures. Further, actigraphy is not the gold standard in evaluating sleep parameters and presents several limitations (De Zambotti et al., 2024). For example, the impossibility of computing a hypnogram unravelling the evolution of sleep phases after long‐haul travels. In the future, the development of EEG wearable devices (e.g., Cannard et al., 2021) could make it possible to obtain this information in competitive settings. Finally, the extent to which our findings may generalize to other sports or disciplines with time changes is not yet known, and additional studies across disciplines are warranted.

### 4.2 Conclusions

Overall, our findings suggest that, when traveling for competitions, accumulated sleep debt and the effects of jet lag in our sample of speed skaters resolve within 5 days. They also underline the importance of providing adequate sleep opportunities and the role of physical activity as a zeitgeber. These findings offer valuable insights for coaches and sports staff in making informed decisions when planning long‐haul travel for competitions. They also point to future research directions. Since short‐track speed skating typically involves intense, repeated efforts, it would be important to explore other sports that involve prolonged continuous efforts to assess the generalizability of these results. (Table 1)

## AUTHOR CONTRIBUTIONS

All authors contributed to the design of the study. François Bieuzen and Evelyne Dubé conducted the experiments, Giorgio Varesco, Chun William Yao, and Evelyne Dubé performed data analysis. Giorgio Varesco performed statistical analysis and wrote the first draft of the manuscript. All authors have read and approved the final version of this manuscript and agree to be accountable for all aspects of the work in ensuring that questions related to the accuracy or integrity of any part of the work are appropriately investigated and resolved. All persons designated as authors qualify for authorship, and all those who qualify for authorship are listed.

## CONFLICT OF INTEREST

None declared.

## Data Availability

Complete step‐by‐step analysis performed on data is available on the Open Science Framework webpage dedicated to the present project (doi: 10.17605/OSF.IO/QSZ9G; link: https://osf.io/qsz9g/?view_only=19a20bf6419340e087342e7818e9678c). The research data will be kept confidential to not compromise ethical standards and legal agreements between the Institut National du Sport du Québec and Speed Skating Canada.

## 1

**TABLE A1:** Model estimates for the effects of time periods on sleep, RAR parameters and neuromuscular performance.

	Baseline		SIA		Race		Travel	
	β ± SE	95% CI	β ± SE	95% CI	β ± SE	95% CI	β ± SE	95% CI
Sleep time (hh:min)	07:09 ± 00:07	06:55; 07:23	07:33 ± 00:06	07:21; 07:46	06:35 ± 00:08	06:19; 06:52	06:16 ± 00:08	06:00; 06:33
Sleep efficiency (%)	81.1 ± 0.8	79.4; 82.8	82.5 ± 0.8	80.9; 84.2	81.6 ± 0.9	79.8; 83.4	80.6 ± 0.9	78.8; 82.5
Bedtime (hh:min)	22:51 ± 00:08	22:35; 23:07	22:33 ± 00:07	22:19; 22:49	00:02 ± 00:09	23:45; 00:21	—	—
Wake‐up time (hh:mm)	07:41 ± 00:08	07:25; 07:56	07:42 ± 00:07	07:28; 07:56	08:08 ± 00:09	07:50; 08:26	—	—
MESOR (AC)	186.3 ± 12.7	159.9; 212.7	178.9 ± 12.1	153.5; 204.4	154.6 ± 13.9	126.2; 183.1	—	—
Acrophase (hh:mm)	13:35 ± 00:20	12:56; 14:14	14:13 ± 00:16	13:42; 14:44	14:08 ± 00:26	13:16; 15:00	—	—
Amplitude (AC)	190.3 ± 12.9	163.6; 217	171.9 ± 12.1	146.5; 197.4	153.7 ± 14.5	124.3; 183.1	—	—
CMJ height (m)	0.41 ± 0.03	0.33; 0.48	0.44 ± 0.03	0.37; 0.51	0.45 ± 0.03	0.38; 0.52	—	—

*Note*: Estimates are calculated using the least‐squares means method. AC, activity counts; SIA, period of stay in Asia.
